# Deconstructing senescence phenotypes in cells of the bone and bone marrow

**DOI:** 10.1172/JCI204645

**Published:** 2026-04-01

**Authors:** Lorenz C. Hofbauer, Martina Rauner

**Affiliations:** 1 Division of Endocrinology, Diabetes and Bone Diseases, Department of Medicine III & University Center for Healthy Aging, Technische Universität Dresden Medical Center, Dresden, Germany.; 2 Center for Regenerative Therapies Dresden, Technische Universität Dresden, Dresden, Germany.

## Abstract

Cellular senescence in osteogenic mesenchymal cells contributes to age-related bone loss. The bone marrow hosts myeloid cells, the precursors of immune cells, as well as mesenchymal cells, which give rise to osteoblasts and osteocytes. The senotype and senolytic response of bone marrow cells, particularly hematopoietic cells, in age-related bone loss is unclear. In this issue, Doolittle et al. showed that of all immune cells, myeloid cells had the strongest senescence profile, yet the relative level of senescence remained lower than that of mesenchymal stromal cells. Mesenchymal cells displayed a profound senotype, rendering them susceptible to senolytic clearance protecting against bone loss. By contrast, selective clearance of p16^+^ myeloid cells was not long-lasting and, hence, did not fully protect against age-related bone loss. These findings underscore the challenges of developing senolytic strategies for tissues with mixed senotypes, such as bone.

## Skeletal cell senescence in health and disease

The appearance of cellular senescence is a hallmark of biological aging across many tissues and organs, including the skeleton (1). Senescent cells are characterized by a distinctive phenotype, termed “senotype,” that includes the activation of cyclin-dependent kinase inhibitors p16^Ink4a^ (p16) and p21^Cip1^ (p21), which leads to DNA double-strand breaks and reflects growth arrest, followed by a senescence-associated secretory phenotype (SASP; i.e., the secretion of a set of pro-inflammatory factors that impair regeneration and initiate tissue fibrosis, as well as matrix-degrading enzymes). In models of skeletal aging, senescent p16^+^ and p21^+^ cells have been detected in the bone/bone marrow compartment in various immune cells and bone cells, including mesenchymal cells, osteochondroprogenitor cells, and osteocytes. Senescence-induced alterations of the bone-building osteoblasts and the mechanosensing osteocytes contribute especially to an impaired anabolic capacity in aging bone, because bone formation under these circumstances can no longer keep up with enhanced bone resorption and essential mechanical cues are not properly sensed or transmitted.

Through a coherent series of studies using cell-specific clearance of senescent cells, investigators from the Mayo Clinic have identified the contribution of cellular senescence as a fundamental principle in the pathogenesis of bone diseases and bone repair. They demonstrated divergent functions for p16^+^ and p21^+^ cells in bone. While p16^+^ osteocytes chiefly contribute to age-related bone loss or bone loss in diabetes or periodontal infection (2–4), p21^+^ osteochondroprogenitors and neutrophils were linked to delays in fracture healing (5). The paper by Saul et al. (5) demonstrated that aged mice display senescent p21^+^ neutrophils and osteochondroprogenitors, two key cellular players in the early phase of bone fracture healing, and that genetic or pharmacologic clearance of senescent cells in murine models accelerates bone fracture healing in both aged and young animals. This study also confirmed the close cooperation of immune and bone progenitor cells in bone repair and highlighted cellular senescence as a modifiable driver of skeletal aging.

## Deconstructing cellular senescence in the bone marrow

In a comprehensive study that appears in this issue of the *JCI*, Doolittle et al. (6) compared the mesenchymal and immune cell senotypes in the bone marrow of aged mice by employing a combination of both global and cell-specific genetic senolytic mouse models with single-cell techniques. Among immune cells within the bone marrow, myeloid-lineage cells, including monocytes and neutrophils, expressed the highest levels of p16^+^ and p21^+^ cells and SASP markers, yet their senescence expression profile was lower than that observed in mesenchymal stromal cells (MSCs). However, since immune cells within the bone marrow by far outnumber MSCs, the senotype expression pattern by myeloid cells may be relevant for skeletal homeostasis in old age.

A major finding of the study was that myeloid cells demonstrated a marked ability to escape senolytic clearance. Senolytic clearance of p16^+^ myeloid cells was induced in a p16-LOX-ATTAC mouse model crossed with the myeloid specific LysM-Cre allele. In this model, ablation of senescent myeloid cells in aged male mice had minimal effects on bone loss that were confined to the metaphysis region of the bone, with intracortical effects; strikingly, ablation had no effect on bone in aged female mice. By contrast, clearance of p16^+^ MSCs mitigated bone loss in both aged male and aged female mice. Extensive studies to delineate the basis for this sexual dimorphism did not reveal a conclusive answer. From various studies it is known that age-related inflammation is higher in male mice and male humans and that estrogen has antiinflammatory effects in the context of aging, though it remains unclear how higher levels of inflammation affect senolytic efficacy.

**Figure 1:**
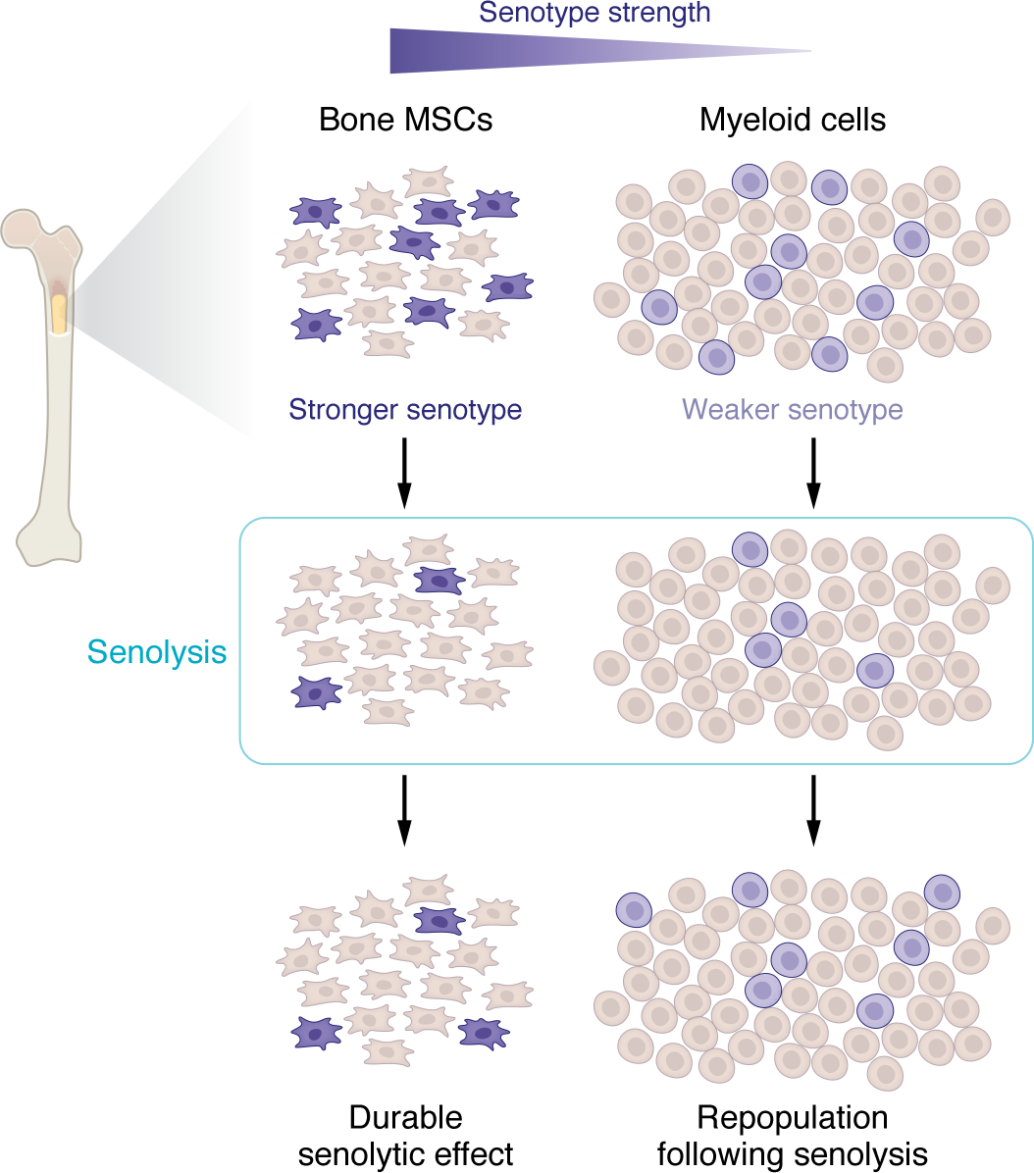
Differences in senotype and senolytic responsiveness between mesenchymal and myeloid cells. Doolittle et al. (6) showed that in bone from aged mice, p16^+^ senescent cells (shown in purple) are frequent in the MSC population and are markedly reduced by senolytic clearance with a long-lasting reductive effect, translating to reduced bone loss. In contrast, cellular senescence is less prominent in myeloid cells. After depletion, senescent myeloid cells display senolytic escape and rapidly replenish, with only mild effects on bone homeostasis.

The main reason for the differences in bone loss outcomes following myeloid versus MSC senolysis was a lack of sustainable clearance of senescent myeloid cells. Although senescent myeloid cells were rapidly cleared in the senolytic model 24 hours after induction, they reappeared shortly thereafter (Figure 1). This may be partially explained by myeloid cells’ shorter life span and higher regenerative potential relative to MSCs, as well as the observation that they displayed only a partial senescence phenotype. For example, the expression of p16^+^ cells in aged mice was 170% higher in mesenchymal cells compared with myeloid cells. It is conceivable that this incomplete senescent phenotype renders them largely refractory to senolytic approaches, whereas the complete senescent phenotype observed in MSCs (6) may be the prerequisite for effective long-lasting senolysis.

Mechanistically, the senotypic difference between myeloid- and mesenchymal-lineage cells may be linked to YAP/TAZ signaling, which integrates mechanical cues to regulate the Hippo pathway, including information about contacts between cells and their extracellular matrix. YAP/TAZ signaling is highly active but declines with age in mesenchymal, but not myeloid, cells (7). When the Hippo pathway is activated, YAP/TAZ are phosphorylated, inhibited, and confined to the cytoplasm, thus making them unable to confer further downstream signals. With inactivation of the Hippo pathway, e.g., via mechanical stimuli, YAP/TAZ remain unphosphorylated and translocate to the nucleus, where they act as effector proteins to control transcriptional coactivators of genes that are involved in cell proliferation and differentiation, thus modulating organ regeneration. In line with this, Doolittle et al. observed that etoposide-induced DNA damage and senescence in MSCs led to a loss of YAP/TAZ activity with upregulation of cGAS/STING signaling, a driver of inflammation and the SASP in these cells (7). In contrast, etoposide did not induce senescence programs in macrophages, which essentially lacked YAP expression and activation. In this sense, YAP/TAZ may represent a key molecular switch that allows cells to initiate senescence programs, which are operative in MSCs but absent in immune cells.

## Future implications of senolytic therapies

Senolytics hold the potential to modify underlying disease mechanisms rather than merely alleviating symptoms, positioning senolytics as a new category of disease-modifying therapies. However, translation of preclinical senescence models into successful senolytic clearance to humans has remained a challenge (8), especially in organ systems that contain a mixed cellular population, such as the bone and bone marrow with its mixture of bone and immune cells that markedly differ in terms of their senotype and consequently their senolytic response. A recent randomized controlled phase II trial explored the established senolytic combination dasatinib/quercetin in 60 postmenopausal women and assessed biochemical markers of bone turnover as study endpoints (9). While the overall study did not meet the endpoints, senolysis with dasatinib/quercetin significantly improved bone markers and increased bone mineral density at the radius in those women with the highest senescent cell burden, determined by p16^+^ T cells (9). This study is consistent with the findings by Doolittle et al. (6) that a certain senescent threshold is required for senolytics to work. An alternative approach to targeting senescent cells is the use of senolytic CAR T cells, which mitigated liver fibrosis and lung cancer in mice (10).

Apart from senolytics, which include dasatinib/quercetin, fisetin, and navitoclax, there are alternative strategies of mitigating the consequences of senescence (8). SASP inhibitors (senomorphics) represent a class of drugs that suppress SASP without eradicating senescent cells and include the antidiabetic drug metformin and the immunosuppressant and mTOR inhibitor rapamycin (8), each with different and undefined risk/benefit ratios. In fact, spurred by media promoting the pursuit of “healthy longevity,” many patients are requesting these drugs. In the context of hematopoietic cells, the difference between SASP and “inflammaging” remains to be defined more clearly. This distinction will also be important in light of the high prevalence of clonal hematopoiesis in aged individuals, which is characterized by inflammation and an impaired differentiation potential of these clones. In the clinic, senomorphics may be particularly useful for chronic, long-term management of age-related diseases characterized by persistent inflammation and tissue dysfunction, including osteoarthritis, atherosclerosis, neurodegenerative disorders, and metabolic disease. By dampening SASP-driven inflammation, senomorphics could improve tissue homeostasis and slow down disease progression without the risks associated with widespread cell clearance. However, key clinical challenges remain, including determining optimal treatment duration, avoiding immunosuppression or metabolic side effects, and identifying biomarkers to monitor SASP modulation in patients. As with senolytics, rigorous clinical trials are needed to define indications, efficacy, and long-term safety.

## Funding support

- LCH and MR by grants from the Deutsche Forschungsgemeinschaft within the Forschungsgruppe-5146 (FerrOs) and SFB/Transregio-369 (DIONE).

## Version 1 04/01/2026

Electronic publication
